# RNA disruption is associated with response to multiple classes of chemotherapy drugs in tumor cell lines

**DOI:** 10.1186/s12885-016-2197-1

**Published:** 2016-02-24

**Authors:** Rashmi Narendrula, Kyle Mispel-Beyer, Baoqing Guo, Amadeo M. Parissenti, Laura B. Pritzker, Ken Pritzker, Twinkle Masilamani, Xiaohui Wang, Carita Lannér

**Affiliations:** Department of Biology, Laurentian University, Sudbury, ON Canada; Department of Chemistry and Biochemistry, Laurentian University, Sudbury, ON Canada; Division of Medical Sciences, Northern Ontario School of Medicine, 935 Ramsey Lake Rd., Sudbury, ON P3E 2C6 Canada; Advanced Medical Research Institute of Canada, Sudbury, ON Canada; Faculty of Medicine, Division of Oncology, University of Ottawa, Ottawa, ON Canada; RNA Diagnostics Inc., Toronto, ON Canada

**Keywords:** Docetaxel, Carboplatin, RNA disruption, Apoptosis, Ovarian tumor cells

## Abstract

**Background:**

Cellular stressors and apoptosis-inducing agents have been shown to induce ribosomal RNA (rRNA) degradation in eukaryotic cells. Recently, RNA degradation in vivo was observed in patients with locally advanced breast cancer, where mid-treatment tumor RNA degradation was associated with complete tumor destruction and enhanced patient survival. However, it is not clear how widespread chemotherapy induced “RNA disruption” is, the extent to which it is associated with drug response or what the underlying mechanisms are.

**Methods:**

Ovarian (A2780, CaOV3) and breast (MDA-MB-231, MCF-7, BT474, SKBR3) cancer cell lines were treated with several cytotoxic chemotherapy drugs and total RNA was isolated. RNA was also prepared from docetaxel resistant A2780DXL and carboplatin resistant A2780CBN cells following drug exposure. Disruption of RNA was analyzed by capillary electrophoresis. Northern blotting was performed using probes complementary to the 28S and 18S rRNA to determine the origins of degradation bands. Apoptosis activation was assessed by flow cytometric monitoring of annexin-V and propidium iodide (PI) binding to cells and by measuring caspase-3 activation. The link between apoptosis and RNA degradation (disruption) was investigated using a caspase-3 inhibitor.

**Results:**

All chemotherapy drugs tested were capable of inducing similar RNA disruption patterns. Docetaxel treatment of the resistant A2780DXL cells and carboplatin treatment of the A2780CBN cells did not result in RNA disruption. Northern blotting indicated that two RNA disruption bands were derived from the 3’-end of the 28S rRNA. Annexin-V and PI staining of docetaxel treated cells, along with assessment of caspase-3 activation, showed concurrent initiation of apoptosis and RNA disruption, while inhibition of caspase-3 activity significantly reduced RNA disruption.

**Conclusions:**

Supporting the in vivo evidence, our results demonstrate that RNA disruption is induced by multiple chemotherapy agents in cell lines from different tissues and is associated with drug response. Although present, the link between apoptosis and RNA disruption is not completely understood. Evaluation of RNA disruption is thus proposed as a novel and effective biomarker to assess response to chemotherapy drugs in vitro and in vivo.

**Electronic supplementary material:**

The online version of this article (doi:10.1186/s12885-016-2197-1) contains supplementary material, which is available to authorized users.

## Background

### Control of cellular RNA degradation

RNA molecules play critical roles in cells, including the facilitation of protein synthesis and regulation of gene expression. To prevent errors in the biosynthesis and function of cellular RNAs, quality control surveillance mechanisms have evolved to identify and preferentially degrade aberrant or nonfunctional RNAs. Degradation of nonfunctional RNAs occurs in regulated stages and involves specialized mechanisms [1]. Mechanisms of controlled RNA degradation exist for messenger RNA (mRNA), transfer RNA (tRNA) and for ribosomal RNA (rRNA) [2–9]. The active components of RNA degradation mechanisms typically include RNA-degrading enzymes or RNases, which interact with numerous co-factors (helicases, polymerases, ubiquitylases, and chaperone proteins) to provide specificity to each type of process.

### Apoptosis and rRNA degradation

The degradation of DNA into internucleosomal fragments is a well-known hallmark of apoptosis [10]. Although not as well-characterized, numerous studies have shown that rRNA may also be degraded, in conjunction with apoptosis, into specific-sized fragments derived from the 28S and/or 18S rRNAs. Early studies demonstrated that both cytotoxic and apoptosis-inducing agents can induce rRNA fragmentation and apoptosis, in a variety of cell lines and types [11–14]. The phenomenon is widespread, also occurring in plants, like oats [15]. Mroczek and Kufel [16] have used the term “stress-induced rRNA fragmentation” to describe the phenomenon by which various cellular stressors activate programmed cell death pathways in yeast, some of which are associated with rRNA degradation. However, the apparent connection between apoptosis and rRNA degradation is not compulsory, as it was shown that rRNA cleavage could occur in the absence of caspase- and BCL-2 dependent apoptosis [17].

### Chemotherapy-dependent “RNA disruption”

Although apoptosis-inducing agents have been shown to induce rRNA degradation in mammalian cells, other cytotoxic agents, such as chemotherapy drugs, have not yet been investigated in this respect. Recently, image-guided tumor core biopsies were taken from patients with locally advanced or inflammatory breast cancer enrolled in the CAN-NCIC-MA.22 clinical trial [18]. Samples were taken prior to, during, and post-treatment with an epirubicin/docetaxel combination chemotherapy. Tumor levels of several biomarkers, including RNA, were then assessed for their relationship to treatment response [18]. Interestingly, a significant association was observed between mid-treatment RNA degradation [19] and the absence of tumor cells in the breast and axilla after treatment (pathologic complete response). The RNA degradation bands were generally of high molecular weight, considerably greater than that observed during autolytic degradation of RNA in tissue samples [19]. These high molecular weight bands were observed following 8–18 weeks of chemotherapy treatment [18]. We refer to this ability of chemotherapy agents to induce long-lived rRNA fragments typically not seen after extensive autolytic degradation as “RNA disruption”. Since the RIN algorithm specifically quantifies the formation of low molecular weight autolytic RNA degradation products [19] and since abnormal RNA banding patterns during RNA disruption results in the assignment of “n/a” values for RIN by the Agilent Bioanalyzer, we recently developed the RNA disruption assay (RDA), which quantifies RNA disruption in tumors and tumor cells as an RNA disruption index (RDI), and is a ratio of abnormal to normal rRNAs [20]. Chemotherapy treatment in the above clinical trial was also found to be associated with reduced tumor RNA content in patients, which may be attributed to both the observed RNA degradation and a suppression of RNA synthesis in tumor cells [20]. Moreover, the above study demonstrated that high tumor RNA disruption mid-treatment was associated with markedly enhanced disease-free survival post-chemotherapy. Our observations of reduced RNA content in patient tumors upon chemotherapy treatment were also consistent with previously published studies indicating that numerous cytotoxic chemotherapeutic drugs can strongly interfere with ribosome biogenesis [21, 22].

In the current study, we describe for the first time an in vitro cell model system for the study of chemotherapy-dependent RNA disruption. This included an investigation into the relationship between RNA disruption, drug type, drug dose, and drug incubation time. We also examined whether RNA disruption reflected the sensitivity of cells to various drugs (previously measured using the clonogenic assay). In addition, we explored the origins of the rRNA fragments and the temporal relationship between the induction of apoptosis by docetaxel, as measured by enhanced caspase activity and annexin V staining, and RNA disruption. Finally, we show that inhibition of caspase-3 activity reduces, but does not eliminate, RNA disruption in response to docetaxel.

## Methods

This study did not require ethics approval from an ethics review committee or board because the study did not involve animals, humans, human data or material directly collected from humans or animals.

### Cell culture

The A2780 parental cell line was acquired from the European Collection of Cell Cultures. The development and characterization of the docetaxel-resistant A2780DXL and carboplatin-resistant A2780CBN cell lines used in this study were described previously [23]. Cell lines were maintained in RPMI-1640 culture medium containing 10 % FBS, 1 % penicillin (10,000 units/ml), and 1 % streptomycin (10,000 μg/ml) (Hyclone Laboratories, Logan, UT, USA). Docetaxel resistance in the A2780DXL cell line was maintained by treating the cells with 0.4 μM docetaxel in complete medium weekly. Carboplatin resistance in A2780CBN cells was similarly maintained by treating the cells with 22 μM carboplatin. The CaOV3 and MCF-7 cell lines, a gift from Dr. Linda Malkas, were maintained in DMEM containing 10 % FBS, 1 % penicillin (10,000 units/ml), and 1 % streptomycin (10,000 μg/ml) (Hyclone Laboratories, Logan, UT, USA). The SKBR3 and BT474 cell lines, a gift from Dr. Robert Lafrenie, were also maintained in DMEM containing 10 % FBS, 1 % penicillin (10,000 units/ml), and 1 % streptomycin (10,000 μg/ml) (Hyclone Laboratories, Logan, UT, USA). The MDA-MB-231 cell line was purchased from the American Type Culture Collection (Manassas, VA, USA) and was maintained in RPMI containing 10 % FBS, 1 % penicillin (10,000 units/ml), and 1 % streptomycin (10,000 μg/ml) (Hyclone Laboratories, Logan, UT, USA). Chemotherapy drugs (docetaxel, paclitaxel, carboplatin, cisplatin, vincristine, etoposide, irinotecan, doxorubicin) were acquired from the pharmacy at Health Sciences North, Sudbury, Ontario, Canada.

### Dose and time exposure experiments

To assess the effect of drug dose on RNA disruption, cells were seeded into six-well plates for 24 h, following which each well was exposed to increasing doses of paclitaxel, docetaxel or carboplatin. After determining the most effective doses to induce RNA disruption, time exposure experiments were performed where cell cultures were exposed to specific drug doses for varying amounts of time (e.g. 24, 48, 72 h). Replicate experiments were performed at least three times.

### RNA isolation and integrity analysis

Cells were harvested by scraping the adherent cells in lysis buffer and collecting them, along with any floating cells by centrifugation. Total RNA was isolated from harvested cells using miRNeasy kits (Qiagen Inc., Toronto, ON, CA). The quantity and integrity of isolated RNA was determined by capillary electrophoresis on an Agilent 2100 Bioanalyzer (Agilent Technologies Canada, Inc., Mississauga, ON, CA) with known reference RNA standards of various masses. The RNA Disruption Index (RDI) was calculated for each sample using a proprietary algorithm (RNA Diagnostics, Inc., Toronto, ON, CA), which computes the ratio of abnormal to normal rRNAs [20].

### Northern blot analysis

Total RNA was isolated from A2780 cells treated with or without 0.2 μM docetaxel for 48 h. The component RNAs (5 μg per lane) were resolved by denaturing formaldehyde 1 % agarose gel electrophoresis, transferred to BioTrace PVDF membranes (Life Sciences, Pensacola, FL, USA), and UV cross-linked. The membranes were then incubated with various radiolabeled probes hybridizing to sequences within the 28S and 18S rRNAs. These probes (Table 1) were derived from rRNA probes described in publications by He et al. [24], Houge et al*.* [12] and Nadano et al*.* [25]. The alignment of all probe sequences were checked against human rRNA sequences (28S rRNA: Genbank ID M11167.1; 18S rRNA: Genbank ID M10098.1) to ensure complete sequence homology. Probes were labeled using γ-^32^P-ATP and the DNA 5’ End Labeling System by Promega (Fisher Scientific, Mississauga, ON, CA). Hybridization was performed according to Brown and Mackey [26]. Following hybridization and washing, blots were sealed in bags and exposed to phosphor imaging screens for various lengths of time. Screens were scanned using a Bio-Rad Molecular Imager FX (Bio-Rad Laboratories, Ltd., Mississauga, ON, CA). Band sizes were determined using Quantity One software from Bio-Rad Laboratories, Inc.

**Table 1:** Oligonucleotide probes for Northern blot analysis of rRNA fragments

Probe	Source	Complementary sequence used in probes	Position in human ^a^28S or ^b^18S rRNA
28SCD1	Houge et al. (1995) [12]	5’-GAC TAA TAT GCT TAA ATT CAG CGG GTC GCC ACG TC-3’	16–50
28SVR2	Nadano et al. (2000) [25]	5’-ACG TGT TAG ACT CCT TGG TCC GTG-3’	1305–1328
28S1	He et al. (2012) [30]	5’-ACC CGG CGT TCG GTT CAT-3’	1867–1884
28S4	He et al. (2012) [30]	5’-GCG GGC CTT CGC GAT GCT TTG TT-3’	3617–3639
28S5	He et al. (2012) [30]	5’-ACC CAG AAG CAG GTC GTC TAC GAA TGG TTT AGC GCC AG-3’	4913–4950
18S1	He et al. (2012) [30]	5’-GCA CCA GAC TTG CCC TCC-3’	698–715
18S2	He et al. (2012) [30]	5’-GAA TAA CGC CGC CGC ATC-3’	1200–1217
18S3	He et al. (2012) [30]	5’-CGG ACA TCT AAG GGC ATC ACA G-3’	1594–1615

^a^28S rRNA sequence Genbank ID M11167.1

^b^18S rRNA sequence Genbank ID M10098.1

### Flow cytometry experiments

To analyze the effect of docetaxel on the proportion of cells entering apoptosis, cells were stained with annexin V and propidium iodide (PI) (CytoGLO Annexin V-FTIC Apoptosis Kit, IMGENEX, San Diego, CA, USA) and the percentage of apoptotic cells (annexin V positive, PI negative) was determined by flow cytometry on a BD FACS Canto II flow cytometer (Becton-Dickinson Biosciences, Mississauga, ON, CA). The effect of docetaxel on cell cycle progression was also assessed by flow cytometry after cells were fixed and stained with PI alone as described previously [27].

### Caspase activity and inhibition assays

Caspase-3 activity in extracts of control and docetaxel-treated cells was assayed by monitoring cleavage of a DEVD substrate using the CPP32 Colorimetric Assay Kit from BioVision Inc. (Milpitas, CA, USA). The effects of caspase-3 inhibition on docetaxel-induced caspase activity and docetaxel-dependent RNA disruption were determined by treating cells with and without docetaxel and/or the caspase-3 inhibitor, Q-DEVD-Oph (BioVision Inc., Milpitas, CA, USA), and then assaying extracts of these cells for caspase-3 activity and RNA disruption as described above.

### Statistical analysis

Statistical analyses were performed using Microsoft Excel or GraphPad Prism 5 software and differences with *p* < 0.05 were considered statistically significant. Significance was determined using a two-way ANOVA with Bonferroni post-hoc test when comparing two variables. A one-tailed t-test was performed to determine the impact of increasing carboplatin concentration on cellular RDI values. For all other data, a two-tailed t-test was performed after application of the F-test to determine equality of variance.

## Results

### Taxane-induced RNA disruption in A2780 and CaOV3 cells is both dose- and time-dependent

**Fig. 1:**
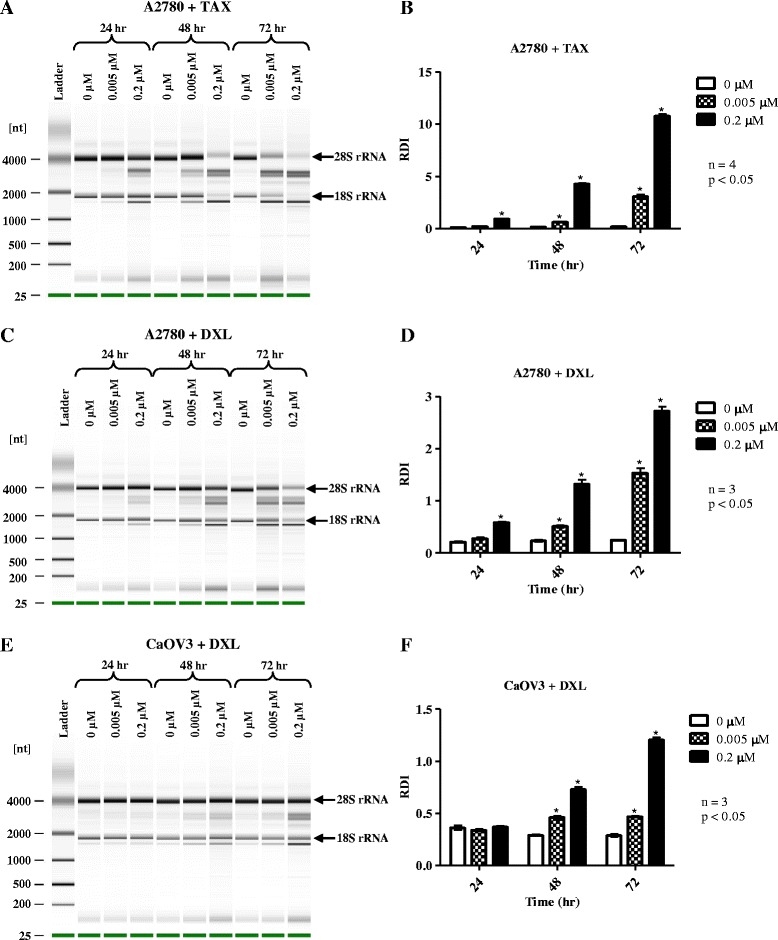
Dose and time-dependent ribosomal RNA disruption in response to taxanes. A2780 and CaOV3 cells were exposed to increasing concentrations of either docetaxel (DXL) or paclitaxel (TAX) for times ranging from 24 to 72 h. Total RNA was isolated from cells following drug exposure and RNA quality was analyzed by capillary electrophoresis. Electropherograms showing RNA mobility were converted to gel images using the Bioanalyzer software. **a** Gel image of RNA from A2780 cells treated with paclitaxel. **b** RDI analysis of RNA isolated from paclitaxel treated A2780 cells. **c** Gel image of RNA from A2780 cells treated with docetaxel. **d** RDI analysis of RNA isolated from docetaxel treated A2780 cells. **e** Gel image of RNA isolated from CaOV3 cells treated with docetaxel. **f** RDI analysis of RNA isolated from CaOV3 cells treated with docetaxel

RNA disruption in A2780 cell cultures became evident at a dose of 0.005 μM for both docetaxel and paclitaxel, but peaked at 0.2 and 1 μM for these drugs, respectively. RNA disruption was evident in the taxane-treated cells by the presence of abnormal bands on the electropherogram, distinct from the normal RNA banding pattern seen in untreated cells (Fig. 1a and c). Furthermore, a significant decrease in total cellular RNA content was also observed upon chemotherapy treatment (Additional file 1). To investigate the effect of time on RNA disruption, A2780 cells were treated with 0.005 or 0.2 μM paclitaxel or docetaxel for 24 to 72 h (Fig. 1a and c). RNA disruption became detectable at 24 h but continued to increase up to 72 h. RDI values confirmed a significant increase in RNA disruption for both paclitaxel- and docetaxel-treated samples over time (Fig. 1b and d). The untreated (0 μM) control sample did not exhibit abnormal bands on electropherograms and retained a low RDI value at all time points.

The CaOV3 ovarian carcinoma cell line was also treated with docetaxel to determine if other ovarian cancer cell lines could exhibit taxane-induced RNA disruption. Using the same docetaxel doses as for A2780 cells, RNA disruption was observed in CaOV3 cells (Fig. 1e) after 48 h of docetaxel treatment and disruption was further increased at 72 h. There was a significant increase in the amount of RNA disruption in both 0.005 and 0.2 μM docetaxel treated CaOV3 cells (Fig. 1f) at 48 and 72 h.

### Carboplatin induces dose dependent RNA disruption in A2780 and CaOV3 cells

**Fig. 2:**
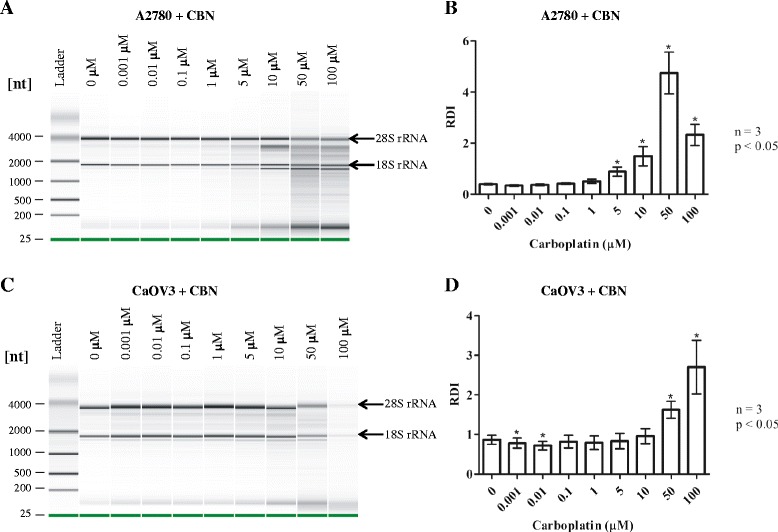
Dose dependence of carboplatin-induced RNA disruption in A2780 and CaOV3 cells. A2780 and CaOV3 cells were treated with increasing concentrations of carboplatin (CBN) for 72 h, the length of time required to detect the RNA disruption response to carboplatin. Total RNA was isolated from cells following drug exposure and RNA quality was analyzed by capillary electrophoresis. **a** Gel image of RNA from A2780 cells treated with carboplatin. **b** RDI analysis of RNA isolated from carboplatin treated A2780 cells. **c** Gel image of RNA from CaOV3 cells treated with carboplatin. **d** RDI analysis of RNA isolated from carboplatin treated CaOV3 cells

Carboplatin, a structurally distinct drug with a very different mechanism of action from taxanes, required a longer exposure time to induce RNA disruption in A2780 and CaOV3 cells. Therefore, A2780 and CaOV3 cells were treated for 72 h with increasing doses of carboplatin. Figure 2a and b display RNA electropherograms and RDI values of A2780 cells treated with 0.001 to 100 μM carboplatin. For A2780 cells, RNA disruption became detectable and significantly higher beginning at 5 μM carboplatin. For CaOV3 cells, RNA disruption was detectable at 10 μM carboplatin but RDI values did not become significantly higher compared to untreated cells until 50 and 100 μM carboplatin were used (Fig. 2c and d).

### Multiple chemotherapy agents induce RNA disruption in breast and ovarian cancer cell lines

**Fig. 3:**
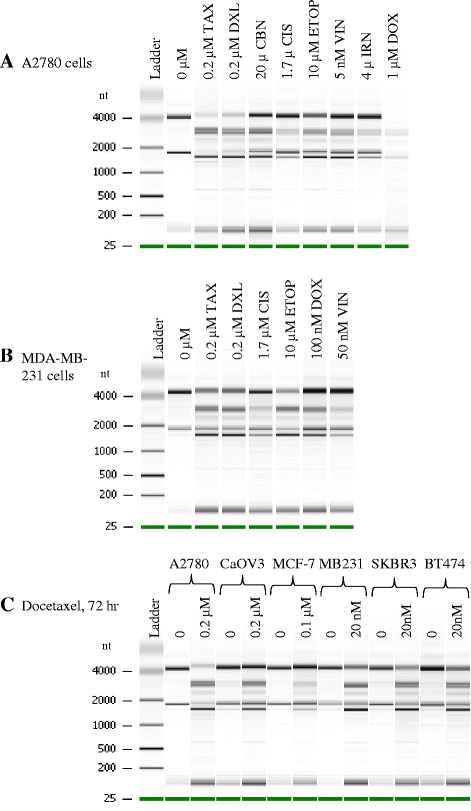
Multiple chemotherapy agents induce RNA disruption in breast and ovarian cancer cell lines. Multiple breast and ovarian cancer cell lines were exposed to various chemotherapy agents and total RNA was isolated from the cells following drug exposure. **a** Gel image of total RNA isolated from A2780 cells treated with multiple chemotherapy agents. All treatments were for 72 h, except carboplatin, which was treated for 120 h. **b** Gel image of RNA isolated from MDA-MB-231, a breast cancer cell line, following treatment with multiple chemotherapy agents. All treatments were for 72 h, except for the control (0 μM) and cisplatin which were treated for 96 h. **c** Gel image of RNA isolated from various breast (MCF-7, MB231, SKBR3, BT474) and ovarian cancer (A2780, CaOV3) cells following 72 h docetaxel treatment, except for MDA-MB-231-0 μM, from which RNA was isolated after 96 h. Abbreviations: TAX-paclitaxel, DXL-docetaxel, CBN-carboplatin, CIS-cisplatin, ETOP-etoposide, VIN-vincristine, IRN-irinotecan, DOX-doxorubicin

To investigate if RNA disruption can be induced by multiple cytotoxic chemotherapy agents, with differing mechanisms of action, A2780 ovarian and MDA-MB-231 breast cancer cells were treated with a panel of chemotherapy drugs. RNA disruption was induced in A2780 cells by treatment with paclitaxel (TAX), docetaxel (DXL), carboplatin (CBN), cisplatin (CIS), etoposide (ETOP), vincristine (VIN), irinotecan (IRN) and doxorubicin (DOX), as shown in the electropherogram in Fig. 3a. In MDA-MB-231 cells, paclitaxel, docetaxel, cisplatin, etoposide, doxorubicin and vincristine were all capable of inducing RNA disruption (Fig. 3b). Finally, chemotherapy drug-induced RNA disruption was observed in multiple breast (MCF-7, MDA-MB-231, SKBR3, BT474) and ovarian (A2780, CaOV3) cancer cell lines following treatment with docetaxel (Fig. 3c), demonstrating that RNA disruption is observed in multiple cell lines of different tissue origin (Figs. 1, 2, 3).

### RNA disruption bands originate from the 28S rRNA

**Fig. 4:**
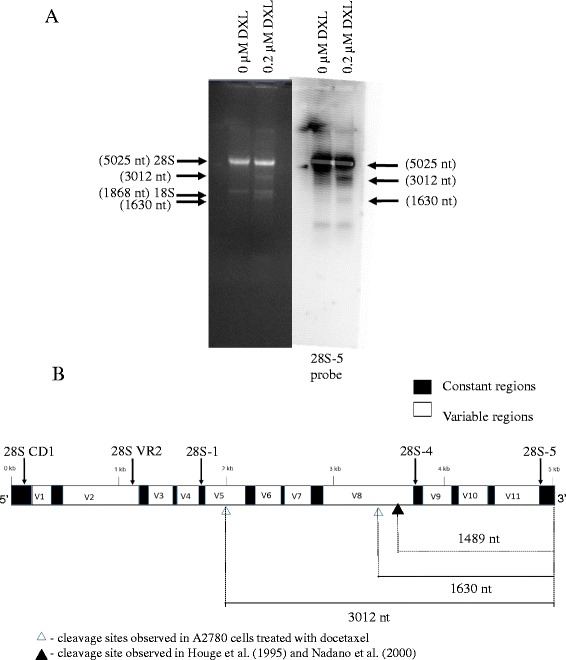
Northern blot analysis of RNA isolated from A2780 cells treated with docetaxel. A2780 cells were treated with 0.2 μM docetaxel for 48 h and total RNA was isolated. RNA was resolved by agarose gel electrophoresis and transferred to PVDF membranes for hybridization with the ^32^P-end-labeled oligonucleotide probe, 28S-5. **a** The panel on the left shows the agarose gel and the panel on the right shows the Northern blot of the gel. RNA bands are indicated by arrows with the size of the band in nucleotides alongside. **b** A schematic diagram of the 28S rRNA sequence showing conserved and variable regions, based on the structure of the 28S rRNA as defined by Gorski et al. (1987) [53] and Wakeman et al. [28]. Location of the probes in the 28S rRNA sequence is shown above the diagram using arrows and the location of the cleavage sites and resulting bands are shown below the diagram

The abnormal RNA disruption bands that occur upon chemotherapy drug exposure are smaller in molecular weight than the 28S and/or 18S rRNAs. To determine whether the abnormal bands originate from the 28S and/or 18S rRNAs, Northern blotting experiments were performed on total RNA prepared from A2780 cells after incubation in the absence or presence of docetaxel for up to 48 h (Fig. 4). Of the probes complementary to the 28S rRNA, only probe 28S-5 hybridized to RNA disruption bands (Fig. 4, Additional files 2 and 3). Figure 4a shows hybridization of the 28S-5 probe to the 28S rRNA and to two smaller bands computed to be 3012 nt and 1630 nt in length. Both of these RNA disruption bands were derived from the 3’end of the 28S rRNA sequence, given the location to which the 28S-5 probe binds within the 28S rRNA (see Discussion). The diagram of the 28S rRNA in Fig. 4b indicates the derivation of the 3012 nt and 1630 nt bands upon RNA disruption. Three probes were used to attempt to detect fragments derived from the 18S rRNA, but none of the probes were able to detect any fragments of the 18S rRNA after 48 h of docetaxel treatment (Additional files 2 and 4).

### RDI values reflect cellular sensitivity or resistance to chemotherapy drugs

**Fig. 5:**
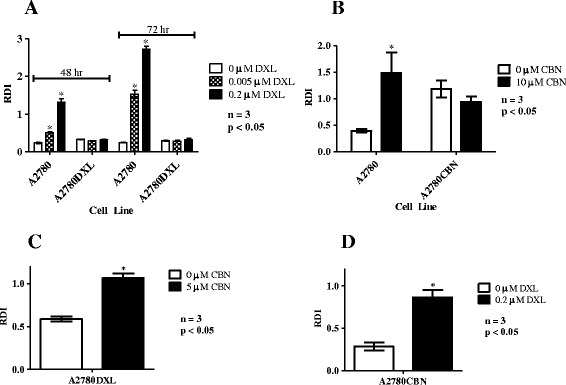
Lack of RNA disruption response in drug resistant cells. A2780 and A2780DXL (resistant to docetaxel) cells were treated with 0, 0.005 and 0.2 μM docetaxel (DXL) for 48 and 72 h. RNA isolated from the cells was analyzed by capillary gel electrophoresis. A2780 and A2780CBN (resistant to carboplatin) cells were treated with 0 and 10 μM carboplatin (CBN) for 72 h. To test for cross-resistance, A2780DXL cells were treated with 0 and 5 μM carboplatin while A2780CBN cells were treated with 0 and 0.2 μM docetaxel. RNA isolated from the cells was analyzed by capillary gel electrophoresis. **a** RDI analysis of RNA isolated from A2780 and A2780DXL cells treated with docetaxel. **b** RDI analysis of RNA isolated from A2780 and A2780CBN cells treated with carboplatin. **c** RDI analysis of A2780DXL cells treated with 0 and 5 μM carboplatin. **d** RDI analysis of RNA isolated from A2780CBN cells treated with 0 and 0.2 μM docetaxel

To investigate the relationship between drug sensitivity and drug-induced RNA disruption, total RNA was isolated from docetaxel-sensitive (A2780) and docetaxel-resistant (A2780DXL) cells after treatment with 0.005 and 0.2 μM docetaxel for 48 or 72 h. Consistent with their known drug sensitivity using clonogenic assays [23], A2780 cells exhibited a significantly higher RNA disruption index than A2780DXL cells did (Fig. 5a). In keeping with the observed RDI values, capillary gel electrophoresis showed docetaxel-induced RNA degradation bands in sensitive A2780 cells but not docetaxel-resistant A2780DXL cells (Additional file 5A). Similarly, RDI values were significantly higher in carboplatin-treated A2780 cells than in similarly treated carboplatin-resistant A2780CBN cells (Fig. 5b). Gel images of the electropherograms showed greater numbers of RNA degradation bands in the A2780 cell line compared to the A2780CBN line (Additional file 5B). In a separate study by our laboratory, Armstrong et al*.* demonstrated lack of cross resistance, using a clonogenic assay, which showed that A2780DXL cells are sensitive to killing by carboplatin and that A2780CBN cells are sensitive to killing by docetaxel [23]. Using RDI analysis we were able to confirm this response, as significantly higher RDI values were observed in the treated resistant cells when compared to the untreated resistant cells, demonstrating sensitivity of the A2780DXL cells to carboplatin and of the A2780CBN cells to docetaxel (Fig. 5c and d). RDA consistently reflected the above differential drug sensitivities, by displaying higher RDI values and RNA disruption bands in drug-sensitive cells (Additional file 5A, B, C, D).

### Concurrent induction of apoptosis and RNA disruption by docetaxel

**Fig. 6:**
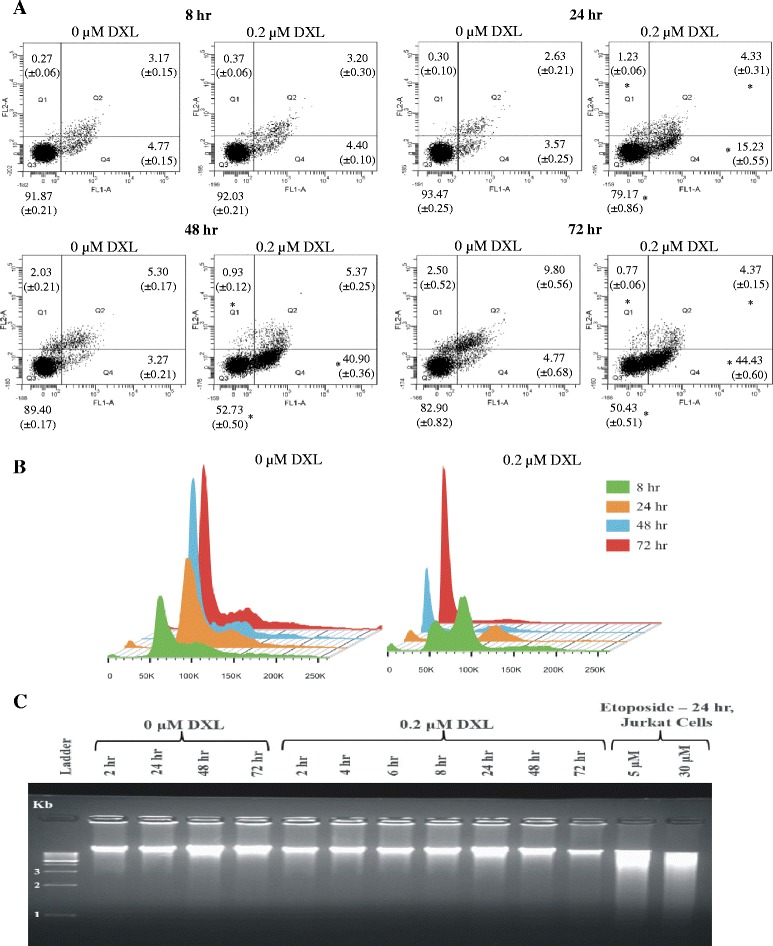
Early and late markers of apoptosis in A2780 cells treated with 0.2 μM docetaxel.A2780 cells were treated with 0.2 μM docetaxel (DXL) for 8, 24, 48 and 72 h. **a** Cells were stained with annexin V-FITC and propidium iodide and analyzed by flow cytometry. Scatter plots of stained cells at each time point are shown. **b** A2780 cells were stained with propidium iodide only and analyzed by flow cytometry. Histograms of stained cells are shown for each time point. **c** DNA laddering in A2780 cells treated with 0 and 0.2 μM docetaxel for times ranging from 2 to 72 h. Jurkat cells were treated with 5 and 30 μM etoposide as a positive control. DNA was isolated from cells at each time point and analyzed by agarose gel electrophoresis. A representative ethidium bromide stained gel is shown

**Fig. 7:**
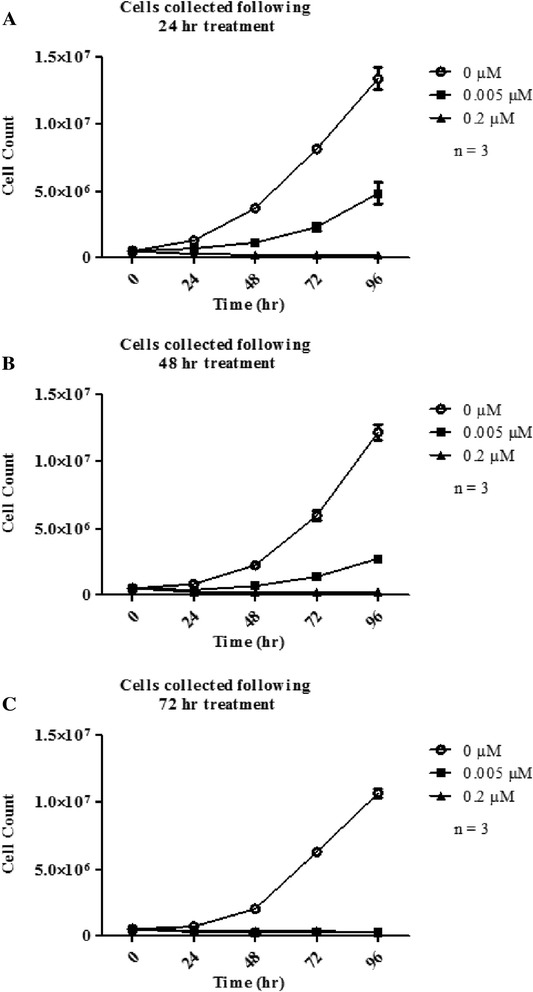
Recovery and proliferation of A2780 cells following docetaxel treatment. In order to assess whether the A2780 cells treated with docetaxel (DXL) that show RNA degradation are able to recover and proliferate, cells were treated with 0, 0.005 or 0.2 μM docetaxel for 24, 48 and 72 h. Following the treatment end point, cells were collected and replated in fresh drug-free medium, and their proliferation was assessed following recovery for 24, 48, 72 and 96 h of replating. **a** Recovery of cells after 24 h treatment. **b** Recovery after 48 h drug treatment. **c** Recovery following 72 h drug treatment

To assess whether docetaxel induces apoptosis and whether this is concurrent with the induction of RNA disruption, A2780 cells were treated with 0.2 μM docetaxel for varying times up to 72 h. Cells were stained with annexin V-fluorescein isothiocyanate (annexin V-FITC) and propidium iodide (PI) and analyzed by flow cytometry (Fig. 6a). At 24 h there was a significant increase in the number of cells stained with annexin V-FITC only, which persisted at 48 and 72 h, indicating that the cells were in early apoptosis. No increase in PI staining was observed up to 72 h, suggesting that cells had retained plasma membrane integrity and had not undergone necrosis. Next, the effect of docetaxel treatment on cell cycle progression was investigated using PI staining of fixed cells following 8, 24, 48 and 72 h of docetaxel exposure (Fig. 6b). A sub G_1_ peak, often associated with apoptotic bodies, was evident in the docetaxel treated cells after 24 h, and by 72 h, almost all the PI signal was in the sub G_1_ peak. This shows that extended docetaxel treatment generated cell fragments with less than a diploid amount of DNA content, representing apoptotic bodies or micronuclei. Finally, to see if DNA laddering (a late apoptosis biomarker) also occurred, A2780 and Jurkat cells were treated with or without docetaxel (A2780 cells) or etoposide (Jurkat cells) for similar lengths of time. Genomic DNA was prepared from the cells and resolved by agarose gel electrophoresis (Fig. 6c). Interestingly, docetaxel-treated A2780 cells did not show any evidence of DNA fragmentation while the DNA from etoposide-treated Jurkat cells was clearly degraded. To confirm that A2780 cells treated with docetaxel were no longer viable despite the lack of DNA degradation, a recovery assay was performed to determine if docetaxel-treated A2780 cells were capable of resuming growth when transferred into drug-free cell culture medium. Following exposure to 0.005 or 0.2 μM docetaxel for up to 72 h, cells were re-plated in fresh, drug-free medium and cultured for up to 96 additional hours (Fig. 7). Cells treated with 0.2 μM docetaxel never recovered (regardless of incubation time), while those treated with 0.005 μM docetaxel could recover after 24 and 48 h of docetaxel exposure but not after 72 h docetaxel exposure. When one relates these observations to the extent of RNA disruption induced in A2780 cells treated with 0.005 or 0.2 μM docetaxel over time (Fig. 1d), it appears that cells can only tolerate a specific level of RNA disruption (RDI = ~0.5), above which cells become nonviable.

### Caspase-3 activation and RNA disruption

**Fig. 8:**
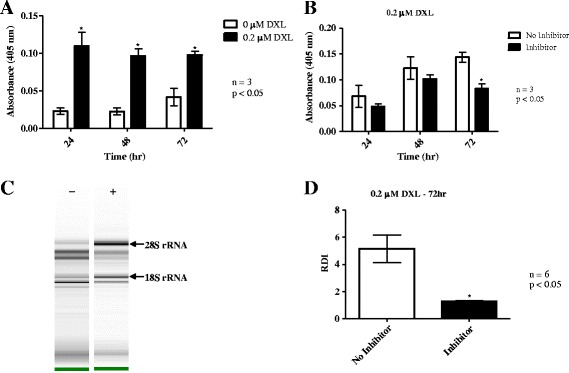
Caspase-3 activation is associated with RNA disruption after docetaxel treatment in A2780 cells. A2780 cells were treated with 0.2 μM docetaxel (DXL) for 24, 48 or 72 h. **a** Caspase-3 activity, assayed using a DEVD substrate, in lysates from A2780 cells. **b** Effect of a caspase-3 inhibitor on caspase-3 activation in lysates from docetaxel treated A2780 cells. **c** Gel image of RNA disruption in A2780 cells treated with docetaxel, in the presence (+) or absence (−) of caspase-3 inhibitor (Q-DEVD-Oph). **d** RDI analysis of RNA from A2780 cells treated with docetaxel for 72 h, with and without the caspase-3 inhibitor

To further support the induction of apoptosis in A2780 cells in response to docetaxel, we examined the effect of docetaxel treatment on caspase-3 activity at 24, 48, and 72 h following treatment. Figure 8a shows that caspase-3 activity increased in response to docetaxel at 24 h and this response persisted through 72 h. The possible connection between docetaxel-induced caspase-3 activation and RNA disruption was then investigated by treating A2780 cells with docetaxel in the absence or presence of the caspase-3 inhibitor (Q-DEVD-Oph). As shown in Fig. 8b, suppression of caspase-3 activity by Q-DEVD-Oph was evident beginning at 24 h after co-treatment with docetaxel, but a statistically significant reduction in caspase-3 activity was only apparent at 72 h (Fig. 8b). Figure 8c depicts the RNA banding pattern for A2780 cells following docetaxel treatment for 72 h, with or without Q-DEVD-Oph. The intact 28S and 18S rRNA bands are indicated by arrows. Without the caspase-3 inhibitor (lane marked -), the original 28S and 18S rRNA bands of untreated cells were reduced in intensity in the presence of docetaxel, while the intensities of the abnormal RNA disruption bands increased. When A2780 cells were co-treated with both docetaxel and Q-DEVD-Oph (lane marked +), the intensities of the 28S and 18S rRNA bands were stronger, while the intensities of the RNA disruption bands were weaker. RDI values for these samples are shown in Fig. 8d, where cellular RNA from docetaxel-treated cells without the caspase-3 inhibitor had a significantly greater RDI value than cellular RNA from docetaxel-treated cells in the presence of the caspase-3 inhibitor, demonstrating that reduced RNA disruption is concomitant with suppression of apoptosis via caspase-3 inhibition.

## Discussion

Previous studies have shown that a variety of apoptosis-inducing agents with distinct mechanisms of action (glucocorticoids, okadaic acid, tumor necrosis factor (TNF), dexamethasone, a calcium ionophore and a tricothecene mycotoxin) were able to induce an ordered, apoptosis-associated rRNA degradation in several different cell types, including plant cells and the unicellular organism yeast [11–13, 15–17, 24]. However, this study is the first to report the ability of structurally distinct cytotoxic chemotherapy agents with different mechanisms of action to induce the formation of high molecular weight rRNA degradation fragments (Figs. 1, 2 and 3), a phenomenon we term “RNA disruption”. This suggests that, despite contrasting structures and mechanisms of action, different chemotherapy agents activate a common rRNA degradation mechanism. Moreover, chemotherapy induced RNA disruption was observed across multiple ovarian and breast cancer cell lines, further supporting the widespread nature of this phenomenon (Fig. 3c).

We further observed that RNA disruption is both dose- and time-dependent (Figs. 1 and 2). Moreover, the extent of RNA disruption closely reflects cellular sensitivity to chemotherapy agents (Fig. 5). RNA disruption was observed to occur only in cell lines demonstrated to have sensitivity to chemotherapy agents (as measured using clonogenic assays) with no RNA disruption being observed in derivative cell lines exhibiting resistance to these agents (Fig. 5). We also show in this study that there is a temporal relationship between induction of apoptosis by docetaxel in A2780 cells and RNA disruption by this agent.

Significant RNA disruption occurred after 24 h exposure to paclitaxel and docetaxel in A2780 cells and after 48 h in CaOV3 ovarian tumor cell lines treated with docetaxel. RNA disruption was always maximal at the longest incubation time (72 h) (Fig. 1). Carboplatin-induced RNA disruption required a longer exposure time for detection (72 h) and RDI values of carboplatin-treated cells were significantly different from untreated cells when a 50 μM drug concentration was applied to both A2780 and CaOV3 cells (Fig. 2). The difference in exposure time and concentration needed to induce RNA disruption may be due to the different mechanisms of action of the two drugs. Furthermore, the drugs produce slightly different rRNA fragments between the 28S and 18S rRNA bands (Figs. 1 and 2), although a common rRNA fragment of slightly greater mobility than the 18S rRNA was generated by both agents. Differing RNA fragmentation patterns were observed by Houge et al*.* depending upon the apoptosis-inducing agent used and the cell line being investigated. They observed a unique cleavage site in the 28S rRNA in bovine endothelial cells, although the RNA cleavage patterns in different cell types were remarkably similar overall [12]. In yeast, different apoptosis-inducing agents or treatments also generated different patterns of rRNA degradation [16]. In another study by He et al*.* in murine macrophages, rRNA cleavage was induced by the tricothecene mycotoxin deoxynivalenol. The pattern observed by He et al*.* contains a similar fragment just below the 18S rRNA and contains three fragments in the region between the 28S and 18S rRNA bands – similar to patterns observed by Houge et al. [12]. In a study by Nadano et al*.,* two different apoptosis-inducing agents were used in Jurkat and U937 cells to initiate rRNA fragmentation (Fas ligand and TNFα), producing a pattern with a band just below the 18S rRNA but with only one clearly discernable band in the region between the 28S and 18S rRNAs [25]. A very different RNA banding pattern was demonstrated by King et al*.* in S49 cells treated with several different apoptotic stimuli. No degradation band below the 18S rRNA was observed and all 5 degradation bands were derived from the 5’-end of the 28S rRNA [17]. In our study, the RNA disruption pattern generated by taxane treatment is the same in the A2780 and CaOV3 cells, composed of a major fragment below the 18S rRNA band and two major fragments between the 28S and 18S rRNA bands (Fig. 1). Carboplatin treatment also generated a fragment just below the 18S rRNA band in both A2780 and CaOV3 cells but appeared to generate multiple fragments in the region between the 28S and 18S rRNA (Fig. 2). Therefore, ordered RNA disruption appears to occur in different ways depending on the cell type and stimulus, suggesting varying mechanisms or reaction kinetics for RNA disruption in cells, depending upon the cell line and/or the RNA disruption-inducing agent.

In this study, we have documented that the ability of specific chemotherapy agents to induce cell death/growth arrest in a clonogenic assay is also reflected in their ability to induce RNA disruption in tumor cells in vitro (Fig. 5). Our data thus suggests that RNA disruption by chemotherapy agents accurately reflects cellular drug sensitivity and drug resistance (as measured using the highly sensitive clonogenic assay). This reinforces the usefulness of the RNA disruption assay (RDA) as a technique for quantifying response to cytotoxic agents such as chemotherapy drugs. Moreover, the RNA disruption assay appears to be equivalent in sensitivity to the clonogenic assay in evaluating response to treatment, is much less labor intensive, is amenable to high throughput approaches, and requires less time to obtain assay results. It may thus represent a valuable tool for drug discovery. The observations of our current study also provide in vitro confirmation of the association of RNA disruption with response to chemotherapy in patients with locally advanced breast cancer [18, 20].

Northern blots of total RNA isolated from A2780 cells treated with docetaxel were used to identify the approximate origins of the RNA disruption bands. As described above, the 28S-5 probe hybridized to the full-length 28S rRNA and to two 28S rRNA fragments – one migrating faster than the full-length 18S rRNA (1,870 nt) and one migrating between the intact 28S (5,025 nt) and 18S rRNA bands (Fig. 4a). Their sizes were determined to be 1,630 nt and 3,012 nt respectively (although these calculated sizes should be interpreted as approximate values). Since both fragments were detected by the most 3’ probe (28S-5), they must have been derived from the 3’-end of the 28S rRNA, with the smaller fragment derived from the larger (Fig. 4b). The size of 3,012 nt for the larger fragment suggests that there must be a cleavage site within the full-length 28S rRNA sequence approximately 2,013 nt from the 5’ end of the molecule (5,025 minus 3,012 nt). The smaller fragment (1,630 nt) indicates another cleavage site located 3,395 nt from the 5’ end of the molecule (5,025 minus 1,630 nt; see Fig. 4b). This interpretation suggests that another 28S rRNA fragment of about 2,013 nt is produced upon docetaxel treatment (2,013 nucleotides from the 5’-end of the molecule), which was not detected by any of the other probes (28S CD1, 28S VR2, and 28S-1). The reason for this is not clear. It is possible that this 2,013 nt fragment, when generated, is not stable and/or is subjected immediately to RNase digestion. In support of this idea, Mroczek and Kufel report that some rRNA fragments are susceptible to digestion by the exosome while other fragments are not [16]. The cleavage site at 3,395 nt from the 5’ end of the 28S rRNA is located in variable region (expansion segment) 8, which has been identified as containing an RNase cleavage site in prior studies [12, 24, 25]. The nucleotide sequence of variable region 8 was correlated by Wakeman et al. with structural features of the human 28S rRNA [28]. Based on this analysis, a double loop structure in the 3’ end of the 28S rRNA contains cleavage sites identified by He et al*.* [24] and Houge et al*.* [12] which are located in the second loop of variable or expansion region 8. The estimated cleavage site in the 3’-end of the 28S rRNA from our data is also located in this loop, supporting the identification of this location as an important site vulnerable to cleavage in response to various cytotoxic stressors. In this study, fragments derived from the 18S rRNA were not detected using three different probes recognizing 18S rRNA sequences (Additional file 4), which indicates that the 18S rRNA was not cleaved within the 48 h period of exposure to docetaxel. Lack of cleavage of the 18S rRNA was reported in several studies, while others show cleavage of both the 18S and 28S rRNAs [11, 12, 17, 24, 25]. These differences in the origins of RNA disruption bands may relate to the agent/stressor inducing rRNA degradation and/or the time of incubation. For example, we have observed the loss of the 18S rRNA band on RNA electropherograms of A2780 cells incubated with taxanes, especially at the later time point of 72 h (Fig. 1a, c).

The degradation of rRNA by cytotoxic stressors has been shown to correlate with the induction of apoptosis in several cell systems [11, 12, 16, 17, 24, 25, 29]. In the current study, the chemotherapy drug docetaxel appeared to induce apoptosis in A2780 cells, as evidenced by elevated annexin V binding (peaking at 72 h; Fig. 6a), increased caspase-3 activity (peaking at 24 h; Fig. 8a), and a dramatic rise in the amount of apoptotic bodies with a sub-G_1_ DNA content (peaking at 72 h; Fig. 6b). RNA disruption was also induced by docetaxel in A2780 cells and was highest at 72 h in this study (Fig. 1). This suggested a temporal correlation between the induction of apoptosis and RNA disruption. The association of RNA disruption with apoptosis is also suggested by our findings that the cell permeable caspase inhibitor Q-DEVD-Oph was able to partially suppress both caspase-3 activity and docetaxel-induced RNA disruption (Fig. 8b, c, and d). This is consistent with previous findings that apoptosis associated rRNA degradation is dependent upon caspase activation [24, 30–34]. However, apoptosis can occur without RNA disruption [35] and rRNA cleavage has been observed in the absence of caspase- and BCL-2 dependent apoptosis [16, 17, 25, 36], suggesting that these two processes can occur independently of each other. Moreover, there is increasing evidence that caspases (such as caspase-3 and caspase-7) may play a role in cell cycle progression independent of their role in promoting cell death [37, 38]. Similarly, the role of caspases in RNA disruption may be unrelated to their role in apoptosis. Our results also show that docetaxel treatment of A2780 cells did not promote DNA laddering (Fig. 6c), a common phenomenon associated with apoptosis in many cell types. However, lack of DNA degradation during apoptosis has been reported in a number of studies [35, 39–41]. Our findings suggest that docetaxel-induced apoptosis in A2780 cells lacks a mechanism for activating DNA fragmentation, while the RNA fragmentation mechanism is not blocked. It is also worth noting that additional studies in our laboratory indicated that apoptotic biomarkers (cleaved caspase-3 and PARP cleavage) wane considerably by 72 h (Additional file 6), while RNA disruption products persist (Figs. 1, 2, and 3). RNA disruption may thus be a preferable biomarker for identifying cells/tissues/patients responding to chemotherapy [18, 20].

At present, the mechanism for chemotherapy-dependent RNA disruption is unknown. A mechanism for rRNA degradation induced by nutritional deficit, called ribophagy, has been described and shown to be distinct from general autophagy [9]. Specific ubiquitination and deubiquitination can protect against or promote ribophagy, indicating an important role for the ubiquitin status of the ribosome in determining susceptibility to ribophagy [9, 42]. However, chemotherapy drug–induced rRNA disruption occurs without any known nutritional deficit and is irreversible after sufficient rRNA disruption has occurred, while ribophagy appears to promote survival during extended nutritional deprivation. A second mechanism for the selective removal of non-functional ribosomes, non-functional rRNA Decay (NRD), has also been described [3, 4, 8, 43]. While ubiquitin also plays a role in NRD, different proteins from those involved in ribophagy appear to be involved, including factors known to act in DNA repair. While we cannot rule out either ribophagy or NRD at this time as mechanisms of ribosomal decay contributing to rRNA disruption, it is worth noting that the generation of specific high molecular weight rRNA fragments has not yet been reported as associated with either ribophagy or NRD.

Another possible mechanism may involve the recently described ability of chemotherapy agents such as docetaxel and doxorubicin to induce TNFα production and release from tumor cells [44]. This could, in turn, result in the activation of a specific receptor for TNFα (TNFR1) known to induce apoptosis through the activation of specific caspases [45, 46]. Consistent with this view, both TNFα and Fas ligand have been shown to induce rRNA degradation in human leukemia cells [25]. Furthermore, chemotherapy agents such as docetaxel and doxorubicin are also well known to induce reactive oxygen species (ROS) in tumor cells, which, in turn, can activate caspases and/or apoptosis [47, 48]. ROS can directly promote the phosphorylation and ubiquitination of Bcl-2 family proteins, resulting in cytochrome c release from mitochondria and caspase activation. Mitochondria appear to be both the source and target of ROS [49–51]. Indeed, Mroczek and Kufel present strong evidence that apoptosis-associated rRNA fragmentation in yeast is correlated with ROS levels, cellular response to oxidative stress, and reduced mitochondrial activity, but not caspase activity [16]. In tumor cells the mechanism could differ somewhat, considering that caspases could be activated by TNFα or ROS generation, resulting in RNA disruption. The evidence provided in the current study demonstrates that a caspase inhibitor significantly reduces docetaxel-induced RNA disruption.

In terms of possible enzymes facilitating RNA disruption, it is highly likely that they are RNA hydrolases (RNases). These RNases could be homologs of RNases recently identified to play a role in stress-induced rRNA degradation in yeast [52]. These investigators identified several RNases associated with stress-induced rRNA fragmentation in yeast, the mammalian homologs of which would be the exonuclease XRN1, an endonuclease involved in rRNA processing (MRP), an RNase associated with the nuclear and cytoplasmic human exosomes (PM/Scl-100), and the mitochondrial nuclease Endonuclease G. Further studies are planned to elucidate the precise mechanism(s) involved in RNA disruption by cytotoxic chemotherapy drugs.

## Conclusions

In this study, we show that RNA disruption can be induced in various cell types by structurally distinct chemotherapy agents with contrasting mechanisms of action in a dose- and time-dependent manner. The disruption fragments (at least for docetaxel) initially stem from cleavages within the expansion segments (variable regions) of the 28S rRNA. The extent of RNA disruption observed in cells reflects their sensitivity (or lack thereof) to chemotherapy drugs and cells can only tolerate a specific level of RNA disruption, above which they become nonviable. Moreover, while the induction of RNA disruption is temporally correlated with the induction of apoptosis, RNA fragmentation products accumulate over time, while apoptotic biomarkers wane. RNA disruption is associated with caspase activation and a known caspase-3 inhibitor can substantially reduce RNA disruption by docetaxel. The link between drug sensitivity in cell lines (previously assessed by clonogenic assay) and drug-induced RNA disruption strongly support our recent clinical findings that high tumor RNA disruption is associated with tumor sensitivity to drug, expressed as a pathologic complete response and enhanced disease-free survival after neoadjuvant chemotherapy in patients with locally advanced or inflammatory breast cancer [20]. RNA disruption thus appears to be a highly reproducible, natural phenomenon observed in tumor cells and represents a powerful new in vitro and in vivo biomarker of chemotherapy response.

## Additional files

Additional file 1: Change in total RNA concentration as a result of paclitaxel treatment. Total RNA was isolated from A2780 cells treated with paclitaxel for 24, 48 and 72 h which were assessed for changes in both, quantity as well as integrity. In addition to the presence of unique bands, a significant decrease in RNA concentration is observed as a result of drug treatment which appears to be both dose and time-dependent. (PPTX 84 kb) Additional file 2: Schematic diagrams of the 28S and 18S human rRNA structures, showing locations of the oligonucleotide probes for the Northern blots. Diagrams of the 28S and 18S rRNA and the location of oligonucleotide probes hybridization used for Northern blot analysis. Regions of conserved sequence and variable (expansion) regions are shown by color blocks in the diagrams. A. Human 28S rRNA structure (5025 nt) with the locations of oligonucleotide probes hybridizing to the 28S rRNA indicated by the arrows. B. Human 18S rRNA structure (1868 nt) with the locations of probes hybridizing to the 18S rRNA are indicated by the arrows. (PPTX 36 kb) Additional file 3: Northern blots of total A2780 RNA hybridized with 28S probes. Panels on the left side show an RNA gel and panels on the right side show corresponding membranes after transfer and blotting. RNA isolated from untreated cells is in lanes marked (-) while RNA isolated from 48 h 0.2 μM docetaxel treated cells is in lanes marked (+). Sizes of RNA bands on gels are shown in nucleotide length (nt) to the left of the figure with arrows indicating each band. Sizes of hybridization targets of probes are shown in nucleotides to the right of the figures with arrows indicating each hybridization target. A. Northern blot results using the 28S-1 probe. B. Northern blot results using the 28S-4 probe. C. Northern blot results using the 28S CD1 probe. D. Northern blot results using the 28S VR2 probe. (PPTX 407 kb) Additional file 4: Northern blots of total A2780 RNA hybridized with 18S probes. Panels on the left side show an RNA gel and panels on the right side show corresponding membranes after transfer and blotting. RNA isolated from untreated cells is in lanes marked (-) while RNA isolated from 48 h 0.2 μM docetaxel treated cells is in lanes marked (+). Sizes of RNA bands on gels are shown in nucleotide length (nt) to the left of the figure with arrows indicating each band. Sizes of hybridization targets of probes are shown in nucleotides to the right of the figures with arrows indicating each hybridization target. A. Northern blot results using the 18S-1 probe. B. Northern blot results using the 18S-2 probe. C. Northern blot results using the 18S-3 probe. (PPTX 410 kb) Additional file 5: RNA disruption response in drug resistant cells treated with chemotherapy agents. The gel images shown in this figure correspond to the RDI data shown in Fig. 5. A. RNA disruption bands occur in the A2780 cells treated with 0.005 μM or 0.2 μM docetaxel (DXL) for 48 or 72 h. The RNA disruption bands are absent in the RNA isolated from the resistant A2780DXL cells treated with either concentration of docetaxel for either 48 or 72 h. B. Gel image of the RNA isolated from A2780 and A2780CBN cells treated with carboplatin, which show the presence of RNA disruption bands in the A2780 cells treated with 10 μM carboplatin for 72 h but display absence of disruption bands in the resistant A2780CBN line. C. Gel image of RNA isolated from A2780DXL cells treated with 5 μM carboplatin for 72 h, showing the presence of unique bands as a result of treatment. D. Gel image of RNA isolated from A2780CBN cells treated with 0.2 μM docetaxel for 48 h, which shows RNA disruption bands. (PPTX 307 kb) Additional file 6: Immunoblots of apoptotic proteins in docetaxel treated A2780 cells. A2780 cells were untreated or treated with 0.2 μM docetaxel for 24, 48, or 72 h. Protein lysates were prepared from the cells, resolved by SDS-PAGE, and transferred to polyvinylidene difluoride (PVDF) membranes. Immunoblotting was performed antibodies against full length caspase 3, cleaved caspase 3, Parp-1 and the loading controls GAPDH and β-actin. Primary antibodies for caspase-3 (3G2), PARP-1 (46D11) and GAPDH (14C10) were from Cell Signaling Technology, Inc. (New England Biolabs, Ltd., Whitby, ON, CA) while HRP-conjugated anti-mouse and –rabbit IgG secondary antibodies were from Santa Cruz Biotechnology, Inc. (Santa Cruz, CA, USA). The Parp-1 antibody detected both full length and cleaved Parp-1. A. Immunoblot showing full length caspase 3. B. Immunoblot showing cleaved caspase 3. B. Immunoblot showing both full length and cleaved Parp-1. C. Immunoblot showing GAPDH. D. Immunoblot showing β-actin. (PPTX 110 kb)

## Abbreviations

ANOVA: analysis of variance
CBN: carboplatin
CIS: cisplatin
DEVD: aspartic acid-glutamic acid-valine-aspartic acid
DMEM: dulbecco’s modified eagle’s medium
DOX: doxorubicin
DXL: docetaxel
ETOP: etoposide
FBS: fetal bovine serum
FITC: fluorescein isothiocyanate
IRN: irinotecan
mRNA: messenger RNA
MRP: multiturnover RNA processing
NRD: nonfunctional rRNA decay
PI: propidium iodide
PVDF: polyvinylidene fluoride
Q-DEVD-Oph: Quinolyl-Asp-Glu-Val-Asp- Difluorophenoxymethylketone
RDA: RNA disruption assay
RDI: RNA disruption index
RIN: RNA integrity number
RNA: ribonucleic acid
RNases: RNA hydrolases
ROS: reactive oxygen species
RPMI: Roswell Park Memorial Institute
rRNA: ribosomal RNA
TAX: paclitaxel
TNF: tumor necrosis factor
TNFR1: tumor necrosis factor alpha receptor 1
TNFα: tumor necrosis factor alpha
tRNA: transfer RNA
UV: ultraviolet
VIN: vincristine
