# Supplementary figures and images for: RNA disruption is associated with response to multiple classes of chemotherapy drugs in tumor cell lines
Source: BMC Cancer. 2016 Feb 24;16:146. doi: 10.1186/s12885-016-2197-1 (PMC4765116; doi:10.1186/s12885-016-2197-1)

## Slide 1
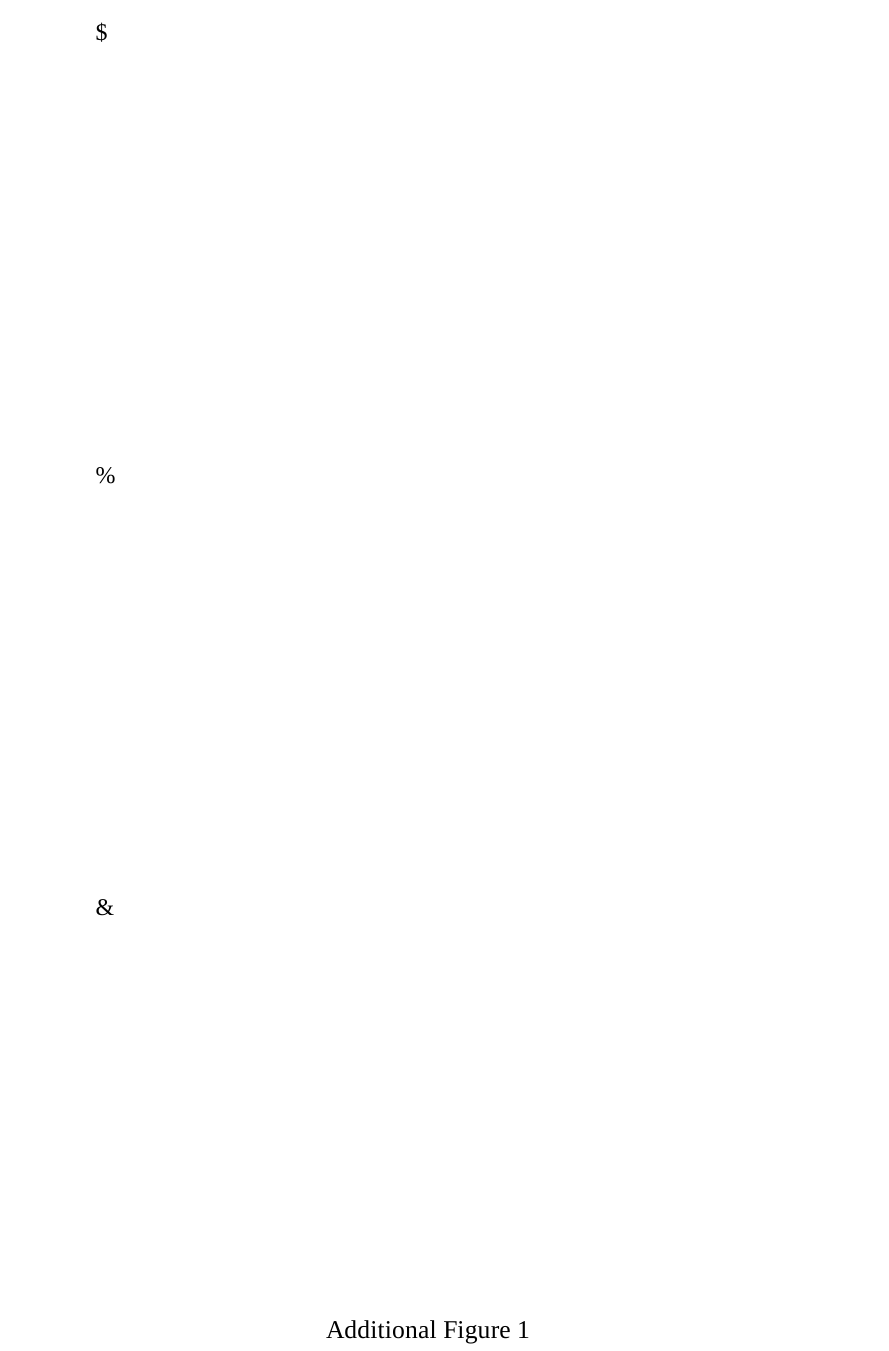

Additional Figure 1

Supplement: Additional file 1: — Change in total RNA concentration as a result of paclitaxel treatment. Total RNA was isolated from A2780 cells treated with paclitaxel for 24, 48 and 72 h which were assessed for changes in both, quantity as well as integrity. In addition to the presence of unique bands, a significant decrease in RNA concentration is observed as a result of drug treatment which appears to be both dose and time-dependent. (PPTX 84 kb) [file 12885_2016_2197_MOESM1_ESM.pptx]

## Slide 1
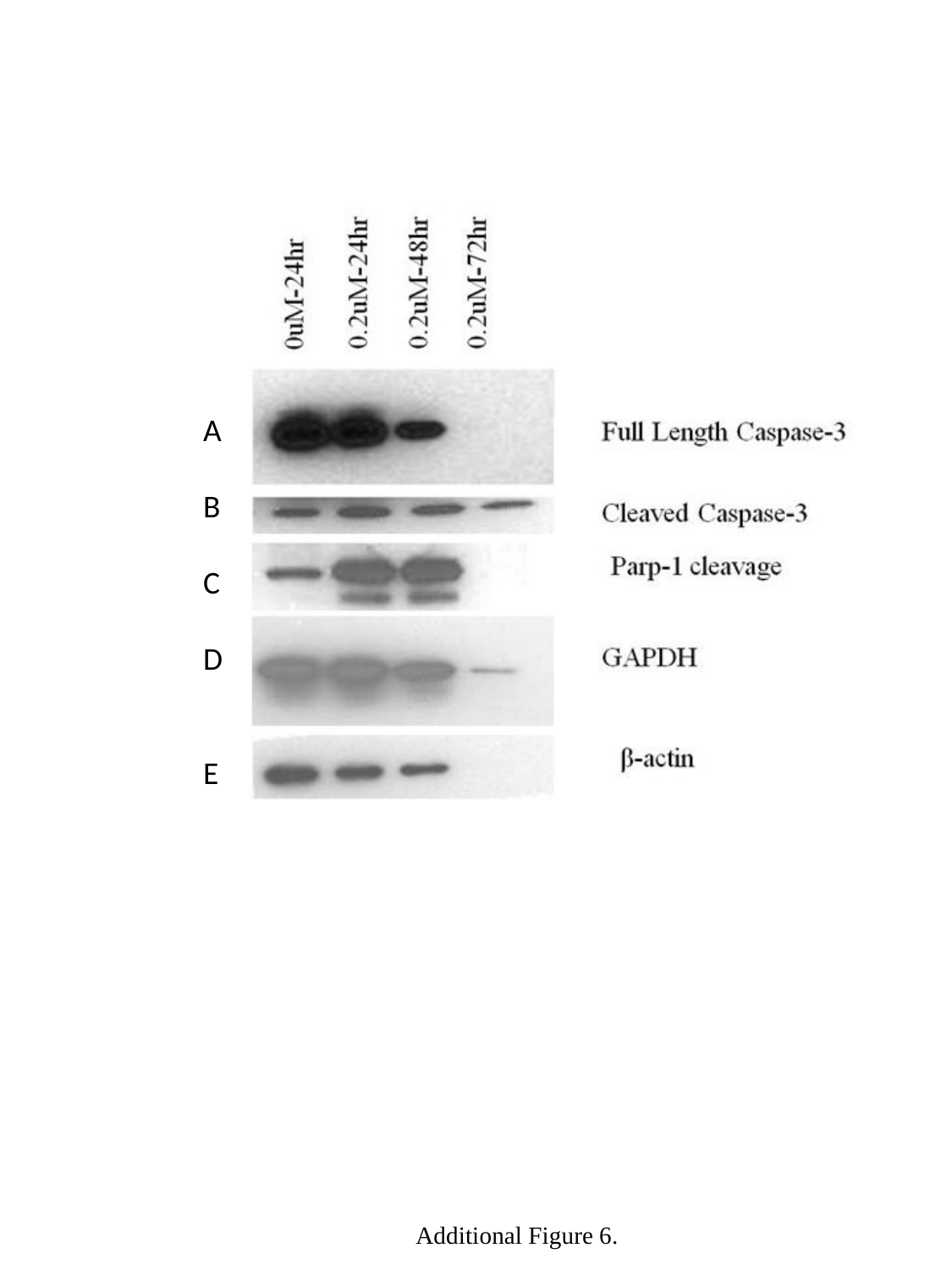

A
B
C
D
E
Additional Figure 6.

Supplement: Additional file 6: — Immunoblots of apoptotic proteins in docetaxel treated A2780 cells. A2780 cells were untreated or treated with 0.2 μM docetaxel for 24, 48, or 72 h. Protein lysates were prepared from the cells, resolved by SDS-PAGE, and transferred to polyvinylidene difluoride (PVDF) membranes. Immunoblotting was performed antibodies against full length caspase 3, cleaved caspase 3, Parp-1 and the loading controls GAPDH and β-actin. Primary antibodies for caspase-3 (3G2), PARP-1 (46D11) and GAPDH (14C10) were from Cell Signaling Technology, Inc. (New England Biolabs, Ltd., Whitby, ON, CA) while HRP-conjugated anti-mouse and –rabbit IgG secondary antibodies were from Santa Cruz Biotechnology, Inc. (Santa Cruz, CA, USA). The Parp-1 antibody detected both full length and cleaved Parp-1. A. Immunoblot showing full length caspase 3. B. Immunoblot showing cleaved caspase 3. B. Immunoblot showing both full length and cleaved Parp-1. C. Immunoblot showing GAPDH. D. Immunoblot showing β-actin. (PPTX 110 kb) [file 12885_2016_2197_MOESM6_ESM.pptx]
