# Supplementary material for: RNA disruption is associated with response to multiple classes of chemotherapy drugs in tumor cell lines
Source: BMC Cancer. 2016 Feb 24;16:146. doi: 10.1186/s12885-016-2197-1 (PMC4765116; doi:10.1186/s12885-016-2197-1)
Supplement: Additional file 2: — Schematic diagrams of the 28S and 18S human rRNA structures, showing locations of the oligonucleotide probes for the Northern blots. Diagrams of the 28S and 18S rRNA and the location of oligonucleotide probes hybridization used for Northern blot analysis. Regions of conserved sequence and variable (expansion) regions are shown by color blocks in the diagrams. A. Human 28S rRNA structure (5025 nt) with the locations of oligonucleotide probes hybridizing to the 28S rRNA indicated by the arrows. B. Human 18S rRNA structure (1868 nt) with the locations of probes hybridizing to the 18S rRNA are indicated by the arrows. (PPTX 36 kb) [file 12885_2016_2197_MOESM2_ESM.pptx]

## Slide 1
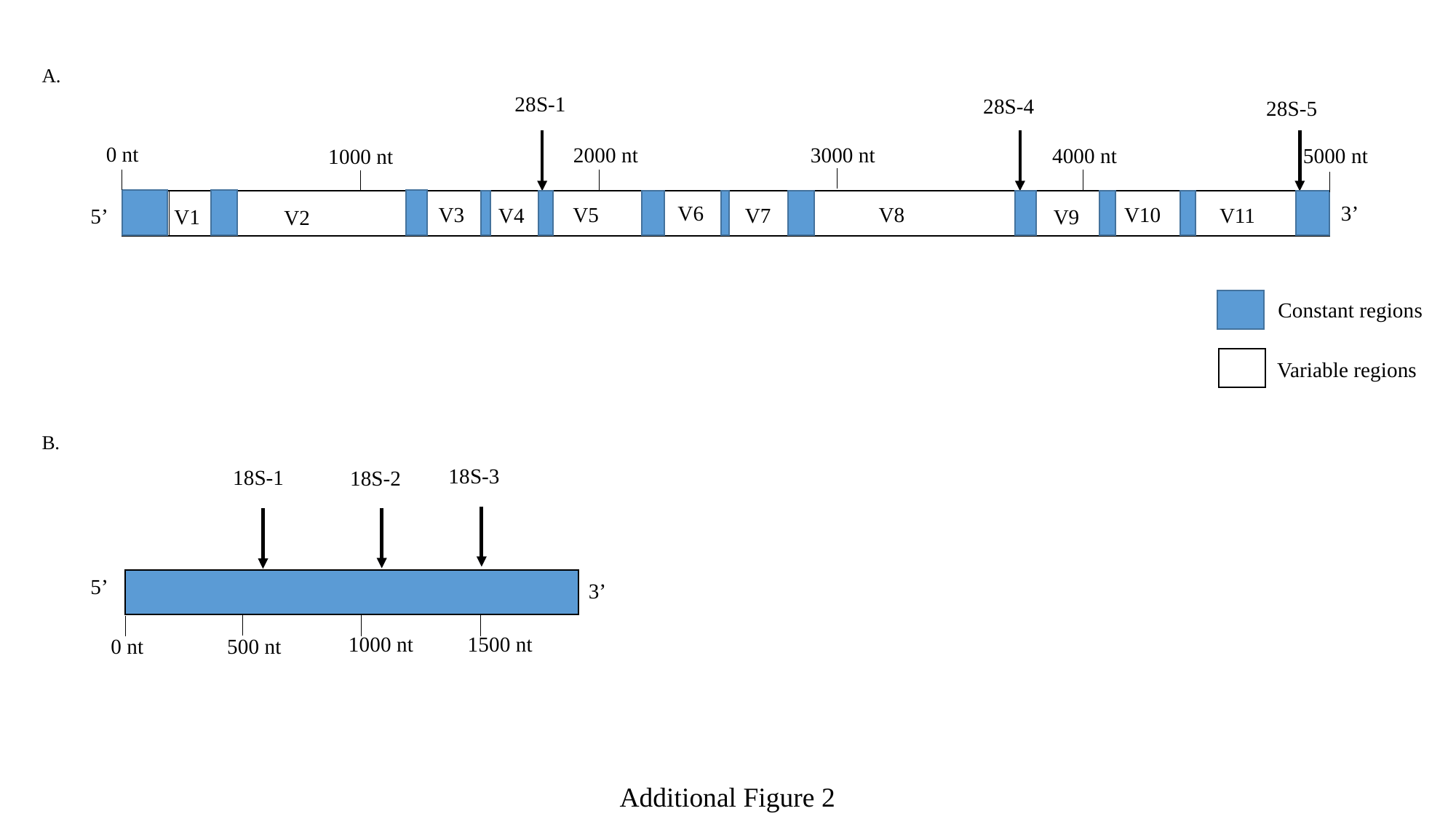

A.
28S-1
28S-4
28S-5
0 nt
2000 nt
3000 nt
4000 nt
5000 nt
1000 nt
V6
V3
V5
V8
V10
V4
V7
V11
V1
V9
V2
3’
5’
Constant regions
Variable regions
B.
18S-3
18S-1
18S-2
5’
1500 nt
1000 nt
500 nt
0 nt
3’
Additional Figure 2
