# Supplementary material for: RNA disruption is associated with response to multiple classes of chemotherapy drugs in tumor cell lines
Source: BMC Cancer. 2016 Feb 24;16:146. doi: 10.1186/s12885-016-2197-1 (PMC4765116; doi:10.1186/s12885-016-2197-1)
Supplement: Additional file 4: — Northern blots of total A2780 RNA hybridized with 18S probes. Panels on the left side show an RNA gel and panels on the right side show corresponding membranes after transfer and blotting. RNA isolated from untreated cells is in lanes marked (-) while RNA isolated from 48 h 0.2 μM docetaxel treated cells is in lanes marked (+). Sizes of RNA bands on gels are shown in nucleotide length (nt) to the left of the figure with arrows indicating each band. Sizes of hybridization targets of probes are shown in nucleotides to the right of the figures with arrows indicating each hybridization target. A. Northern blot results using the 18S-1 probe. B. Northern blot results using the 18S-2 probe. C. Northern blot results using the 18S-3 probe. (PPTX 410 kb) [file 12885_2016_2197_MOESM4_ESM.pptx]

## Slide 1
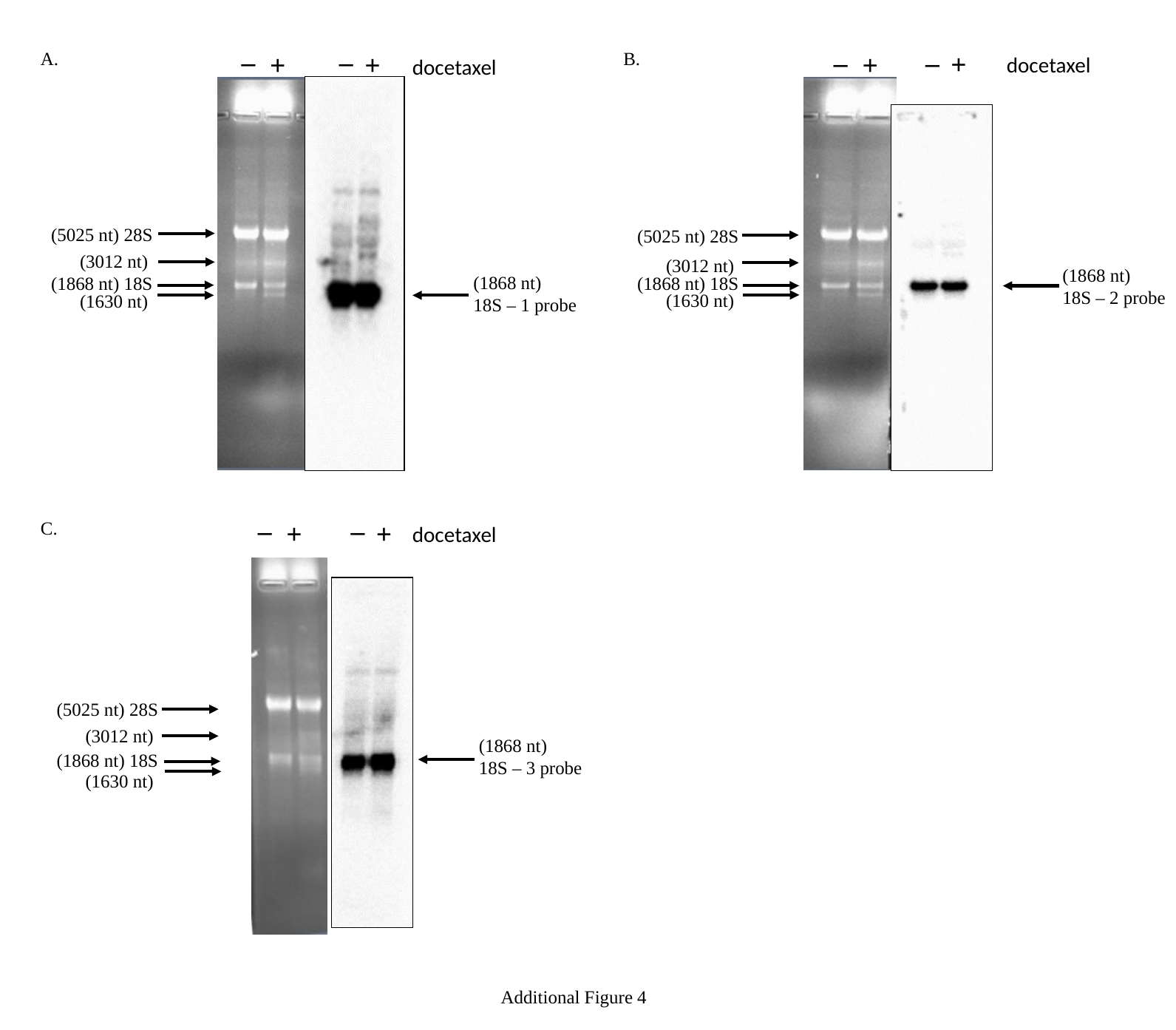

–
–
+
+
(5025 nt) 28S
(3012 nt)
(1868 nt)
18S – 1 probe
(1868 nt) 18S
(1630 nt)
–
–
+
+
(5025 nt) 28S
(3012 nt)
(1868 nt)
18S – 2 probe
(1868 nt) 18S
(1630 nt)
A.
B.
docetaxel
docetaxel
–
–
+
+
(5025 nt) 28S
(3012 nt)
(1868 nt)
18S – 3 probe
(1868 nt) 18S
(1630 nt)
C.
docetaxel
Additional Figure 4
