# Supplementary material for: RNA disruption is associated with response to multiple classes of chemotherapy drugs in tumor cell lines
Source: BMC Cancer. 2016 Feb 24;16:146. doi: 10.1186/s12885-016-2197-1 (PMC4765116; doi:10.1186/s12885-016-2197-1)
Supplement: Additional file 5: — RNA disruption response in drug resistant cells treated with chemotherapy agents. The gel images shown in this figure correspond to the RDI data shown in Fig. 5. A. RNA disruption bands occur in the A2780 cells treated with 0.005 μM or 0.2 μM docetaxel (DXL) for 48 or 72 h. The RNA disruption bands are absent in the RNA isolated from the resistant A2780DXL cells treated with either concentration of docetaxel for either 48 or 72 h. B. Gel image of the RNA isolated from A2780 and A2780CBN cells treated with carboplatin, which show the presence of RNA disruption bands in the A2780 cells treated with 10 μM carboplatin for 72 h but display absence of disruption bands in the resistant A2780CBN line. C. Gel image of RNA isolated from A2780DXL cells treated with 5 μM carboplatin for 72 h, showing the presence of unique bands as a result of treatment. D. Gel image of RNA isolated from A2780CBN cells treated with 0.2 μM docetaxel for 48 h, which shows RNA disruption bands. (PPTX 307 kb) [file 12885_2016_2197_MOESM5_ESM.pptx]

## Slide 1
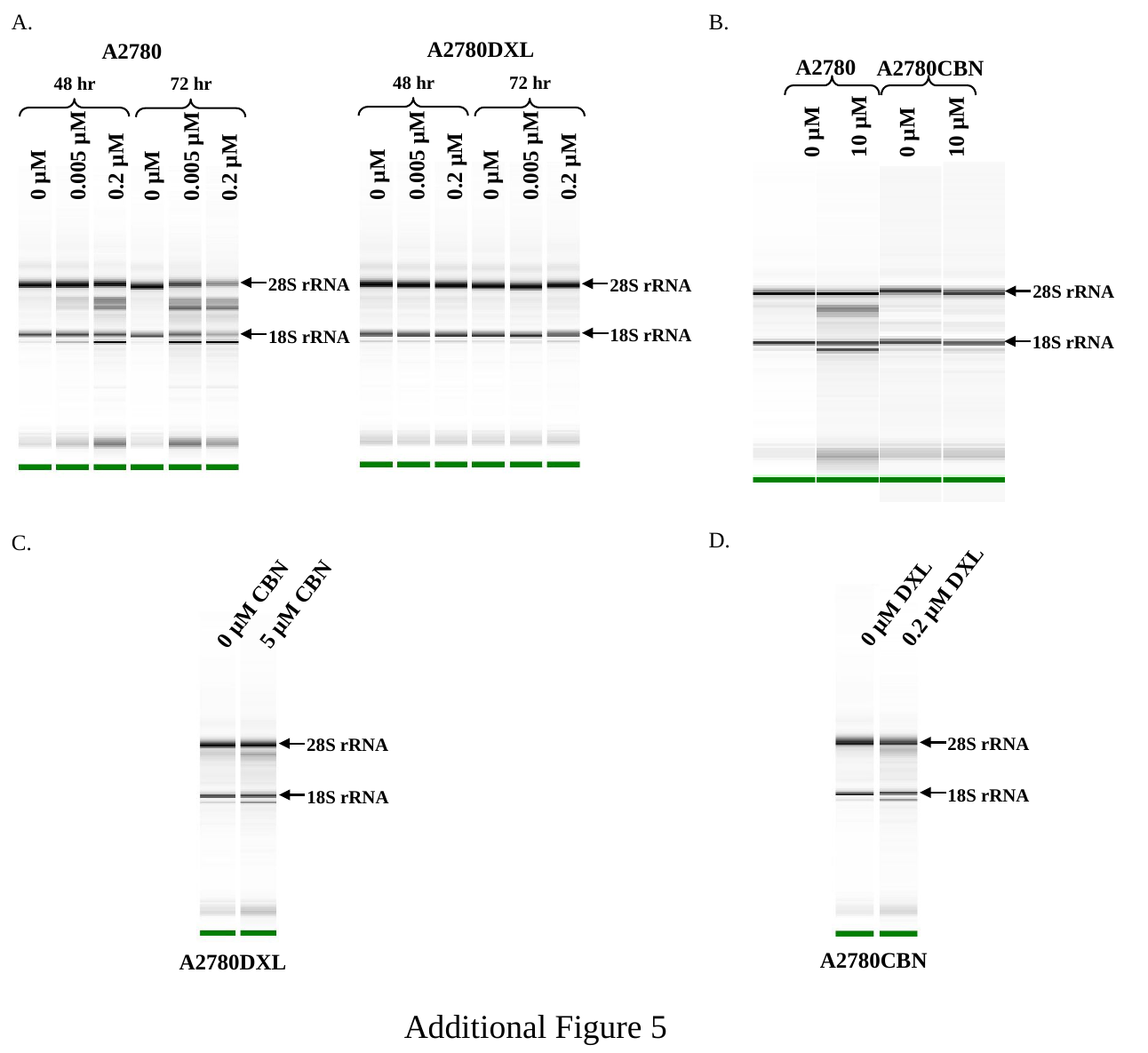

A.
B.
A2780DXL
A2780
A2780
A2780CBN
48 hr
72 hr
48 hr
72 hr
0 µM
10 µM
10 µM
0 µM
0.005 µM
0.2 µM
0 µM
0 µM
0 µM
0.005 µM
0.2 µM
0.005 µM
0.2 µM
0.005 µM
0.2 µM
0 µM
28S rRNA
28S rRNA
28S rRNA
18S rRNA
18S rRNA
18S rRNA
D.
C.
0 µM DXL
0.2 µM DXL
A2780CBN
0 µM CBN
5 µM CBN
A2780DXL
28S rRNA
28S rRNA
18S rRNA
18S rRNA
Additional Figure 5
